# Crossing Cultures, Gaining Weight? A Multidimensional Analysis of Health Behaviors in Chinese Students Overseas

**DOI:** 10.3390/healthcare13212804

**Published:** 2025-11-04

**Authors:** Xiao-Lin Wen, In-Whi Hwang, Jun-Hao Shen, Ho-Jun Kim, Kyu-Ri Hong, Jung-Min Lee

**Affiliations:** 1 Department of Physical Education, Graduate School of Physical Education, Kyung Hee University, Yongin-si 17104, Republic of Korea; ssdlh0422@khu.ac.kr (X.-L.W.); 2022310074@khu.ac.kr (J.-H.S.); wnsghrla1@khu.ac.kr (H.-J.K.); 2 Department of Sports Medicine and Science, Graduate School of Physical Education, Kyung Hee University, Yongin-si 17104, Republic of Korea; inn95313@khu.ac.kr; 3 Department of Physical Education, Graduate School of Education, Kyung Hee University, Yongin-si 17104, Republic of Korea; rbfl2526@khu.ac.kr; 4 Department of Physical Education, Kyung Hee University, Yongin-si 17104, Republic of Korea

**Keywords:** weight change, physical activity, sedentary behavior, psychological stress, international students, Chinese students

## Abstract

**Background/Objectives**: This study investigates the multifactorial determinants of weight change among Chinese international students in South Korea, focusing on physical activity (PA), sedentary behavior (SB), sleep quality, and psychological stress. **Methods**: Data were collected from 445 Chinese international students (male = 224, 50.3%) using self-administered questionnaires and follow-up interviews. Participants were categorized into weight gain and weight loss groups based on changes in body weight and BMI. Multinomial logistic regression was used to examine the relationships between lifestyle factors and weight change. **Results**: The reference group consisted of males and females in the weight loss group. Weight gain was more likely in males experiencing frequent depression (OR = 1.84, *p* < 0.001), while frequent stress decreased the likelihood of weight gain (OR = 0.24, *p* < 0.01). Males with weight gain were more likely to experience frequent fatigue (OR = 1.24, *p* < 0.05) and engage in optimal moderate PA (OR = 1.98, *p* < 0.05). In females, weight gain was less likely with frequent fatigue and high-intensity PA (OR = 0.25, *p* < 0.05). Conversely, weight gain was more likely in females with optimal moderate PA and reduced sleep duration (OR = 1.68, *p* < 0.05; OR = 2.28, *p* < 0.01). **Conclusions**: This study identifies gender-specific effects of mental health, PA, SB, and sleep patterns on weight changes among Chinese international students. These findings highlight the need for targeted health strategies addressing mental health, PA, and sleep to support weight management, particularly in international student populations.

## 1. Introduction

With the rapid acceleration of globalization, an increasing number of students are pursuing higher education abroad to broaden their perspectives and acquire cross-cultural insights [1]. According to the 2023 report by the Korean Ministry of Education, the number of international students enrolled in higher education institutions in Korea reached 181,842 by the end of the year, with Chinese students comprising 68,065, or over one-third of the total population [2]. As China remains a leading source of international students, attention has turned to the unique health-related challenges this population faces during their academic sojourns, notably weight fluctuations [3,4,5]. These weight changes may adversely affect physical health, psychological well-being, and academic performance, emphasizing the need to identify the underlying determinants to promote healthier lifestyle adaptations [6].

Weight trajectories among international students are influenced by environmental factors that can vary considerably across and within host countries [7]. For example, students enrolled in universities situated in regions with high obesity prevalence are more likely to experience weight gain [8]. Barriers such as limited language proficiency and cultural unfamiliarity may hinder participation in local sports or physical activity (PA), further contributing to sedentary lifestyles [9]. These weight fluctuations, in turn, are associated with both perceived body image and objective health outcomes [10,11]. Excessive weight gain has been linked to increased risks of metabolic disorders, including type 2 diabetes, hypertension, and cardiovascular disease, whereas underweight status is associated with malnutrition, compromised immune function, and reduced quality of life [12,13,14].

Engagement in regular PA serves as an effective strategy for both the prevention and management of chronic diseases such as cardiovascular and metabolic disorders, musculoskeletal conditions, cancer, and mental health issues [15,16,17]. However, international students often experience constraints on PA due to demanding academic schedules and sociocultural adjustments, which limit opportunities for regular exercise [18].

Concurrently, rapid technological advancements and shifting lifestyle patterns have led to a rise in sedentary behavior (SB), defined as waking activities involving ≤1.5 metabolic equivalents (METs) performed in seated, reclining, or standing positions [19]. SB is associated with multiple adverse health outcomes [20]. These behaviors are broadly categorized into screen-based (e.g., television, smartphones, gaming) and non-screen-based (e.g., reading, paperwork) activities [21]. Although such behaviors are embedded in modern academic life, they have been implicated in poor metabolic outcomes and are particularly pervasive among university students due to both academic and leisure-time screen exposure [22]. The displacement of PA by SB is a significant contributor to weight gain in this population [23,24].

Another critical factor influencing weight change is sleep quality, which is often compromised due to acculturative stress—a constellation of psychological and social stressors including discrimination, homesickness, cultural dissonance, and adjustment-related anxiety [25]. Such stress is associated with sleep disturbances, which may further exacerbate metabolic dysregulation and hormonal imbalances [26,27,28]. Consequently, poor sleep quality may contribute to both weight gain and loss.

Psychosocial stress does not affect eating uniformly. Under stress, some individuals increase intake (hyperphagia) often preferring energy dense, highly palatable foods whereas others reduce intake (hypophagia). The direction and magnitude of change appear to vary by stressor characteristics (e.g., intensity, duration) and individual traits such as emotional-eating tendency, dietary restraint, and reward sensitivity. International students often experience heightened levels of anxiety and depression related to academic pressures and social isolation [29]. These psychological disturbances can disrupt appetite regulation, resulting in either hypo- or hyperphagia, thereby influencing body weight [30,31]. Thus, psychological stress, in conjunction with lifestyle behaviors, plays a critical role in shaping health outcomes among international students [32].

While prior research has examined associations between weight gain and general health-related behaviors among international students [33]. In Korea, Chinese international students face a distinct acculturation context language and cultural adaptation, new campus food environments, and shifts in daily routines and social networks that can differentially shape 24 h movement behaviors and psychosocial stress. There is limited empirical evidence specifically addressing the combined roles of PA, SB, sleep quality, and psychological stress in Chinese international students [34,35,36]. To address this gap, the present study aims to comprehensively investigate the multifactorial determinants of weight change through the lens of 24 h movement behaviors and psychosocial factors in Chinese international students. This research seeks to inform targeted intervention strategies and public health initiatives tailored to this high-risk and rapidly growing population.

## 2. Materials and Methods

### 2.1. Study Design and Participants

This study employed a cross-sectional survey design to investigate factors associated with weight changes among Chinese international students residing in South Korea. Data collection was conducted through a structured, self-administered questionnaire and follow-up telephone interviews. The survey instrument included sections assessing demographic characteristics, PA time, SB time, sleep characteristics, and mental health variables. This study was reviewed and approved by the Kyung Hee University Institutional Review Board (IRB: KHGIRB-25-341 on 10 July 2025). Written informed consent was obtained from all participants prior to data collection; no waiver of consent was requested or granted. All data were de-identified before analysis to ensure confidentiality.

To minimize regional bias and ensure diversity, participants were recruited from over ten universities across South Korea through online survey distribution and individual interviews. Eligibility criteria included: (1) Chinese nationality, (2) enrollment in a South Korean university for a minimum of six months, and (3) current residence in South Korea. A total of 658 students participated, with 345 males (52.45%). After applying the exclusion criteria, 199 participants were removed. Of these, 89 were removed due to incomplete responses, 69 were excluded because of response inconsistencies suggesting potential bias, and 55 were removed for missing key variables such as sex, age, PA, or SB time. The final sample consisted of 445 students, including 224 males (50.34%).

### 2.2. Group

Weight change was calculated as current minus previous weight. For the primary analysis, we classified gain if Δweight > 0 and loss if Δweight < 0. Previous (“baseline”) weight was self-reported at an event-anchored timepoint: “What was your body weight (kg) during the two weeks before leaving for Korea?” To enhance accuracy, participants were reminded that a pre-departure medical examination is commonly required for enrolment visa procedures; where available, they were asked to consult the recorded weight from that examination when answering. Current weight was self-reported in kilograms.

Participants were classified into four groups based on changes in body weight and body mass index (BMI) following immigration. Specifically, individuals whose current weight and BMI were higher than their pre-immigration values were placed in the weight gain group, while those whose current weight and BMI were lower were placed in the weight loss group. The sample was divided into four subgroups: males with weight gain (*n* = 141), males with weight loss (*n* = 83), females with weight gain (*n* = 131), and females with weight loss (*n* = 90).

Physical activity (PA): Weekly minutes were computed as days × minutes/session for moderate physical activity (MPA) and vigorous physical activity (VPA). Categories were defined as: Insufficient (<150 min/week MPA and <75 min/week VPA), Optimal (meeting either ≥150 min/week MPA or ≥75 min/week VPA), and High (exceeding ≥300 min/week MPA or ≥150 min/week VPA, or the equivalent combination).

Sedentary behaviour (SB): Average daily sitting time was derived from weekday (×5) and weekend (×2) reports. Categories: Low (<5 h/day), Normal (5–8 h/day), Excessive (>8 h/day). SB was defined as waking behaviour in a sitting, reclining, or lying posture with ≤1.5 METs; prolonged standing was not considered sedentary.

Sleep: Self-reported average sleep duration was grouped as Short (<7 h/day), Normal (7–9 h/day), Long (>9 h/day).

### 2.3. Measures

#### 2.3.1. Demographics and Socioeconomic Indicators

Data were collected on sex, age, residential status, type of housing (e.g., dormitory, apartment, detached house), and satisfaction with living environment, measured on a five-point Likert scale ranging from 1 (very dissatisfied) to 5 (very satisfied).

#### 2.3.2. Lifestyle Behaviors

Smoking status was categorized as current, former, or never. Alcohol consumption was classified into six categories, ranging from “none in the past year” to “4 or more times per week.”

#### 2.3.3. Physical Activity and Sedentary Behavior

The International Physical Activity Questionnaire (IPAQ) was used to assess weekly PA, including moderate and vigorous intensity PA, and SB [37,38]. PA duration was computed by multiplying the number of days by the average time per session. Sedentary time was assessed separately for study and leisure purposes on weekdays and weekends. Weekly totals were derived by multiplying weekday values by five and weekend values by two.

#### 2.3.4. Sleep Characteristics

Sleep duration and quality were assessed using the Sleep Timing Questionnaire (STQ) [39]. Items captured average daily sleep on weekdays and weekends, frequency of late-night activity, perceived sleep deprivation, and fatigue relief after compensatory sleep.

#### 2.3.5. Acculturative Stress and Mental Health

Acculturative stress was assessed using items from the Acculturative Stress Scale for International Students (ASSIS) and the Migration-Acculturative Stressor Scale [35,40,41]. These instruments measured stress related to language barriers, discrimination, cultural adaptation, academic pressures, and social isolation. Fatigue, stress frequency, and self-reported depression were assessed on a 4-point Likert scale: (1) frequently, (2) occasionally, (3) rarely, (4) never.

#### 2.3.6. Questionnaires (Chinese Versions and Adaptation)

All three instruments were administered in Chinese, as follows: (i) the IPAQ (short, self-administered) was downloaded from the official IPAQ repository and scored using the 2005 combined protocol; (ii) the Sleep Timing Questionnaire (STQ) does not have an official or published Chinese form, so sleep timing was measured using a Chinese adaptation developed via forward–back translation, expert-panel reconciliation, and cognitive debriefing with Chinese international students; and (iii) the Acculturative Stress Scale for International Students (ASSIS) Chinese version, previously validated in Chinese student samples. No wording changes were made to the IPAQ or ASSIS beyond formatting for online delivery.

### 2.4. Covariates

Covariates of age and sex were included to control for potential confounding in the relationship between health-related factors and weight change among Chinese international students. Age (in years) was calculated from self-reported birthdates, while height (without shoes) and weight (without heavy clothing) were measured and used to compute body mass index (BMI, kg/m^2^). Given the paucity of prior research on weight changes before and after studying abroad, these covariates were chosen for their roles as fundamental demographic factors and possible mediators or confounders, rather than solely on precedent. All data were obtained via self-report questionnaires, and statistical models were adjusted for age and sex to account for their influence on weight trajectories during the study abroad period.

### 2.5. Data Analysis

All statistical analyses were conducted using SPSS version 28.0 (SPSS Inc., Chicago, IL, USA). Descriptive statistics were used to summarize sample characteristics. The chi-square analysis (χ^2^ test) was used to compare the categorical data of general characteristics among weight change status. To assess the associations between behavioral and psychosocial predictors and weight change categories, multinomial logistic regression analysis was used. Results were reported as odds ratios (OR) with 95% confidence intervals (CI). Statistical significance was set at *p* < 0.05.

## 3. Results

Table 1 summarizes the changes in weight and BMI among participants (*n* = 445) before and after studying abroad. The cohort was evenly distributed by gender, comprising 224 males (50.3%) and 221 females (49.7%). The results reveal a significant increase in both weight and BMI for both genders after studying abroad. Among males, the average weight increased from 70.0 ± 11.7 kg to 72.4 ± 12.5 kg, while BMI rose from 22.2 ± 3.6 kg/m^2^ to 23.0 ± 3.5 kg/m^2^. Similarly, females experienced an increase in average weight from 56.9 ± 9.9 kg to 58.0 ± 10.7 kg, with BMI increasing from 20.7 ± 3.2 kg/m^2^ to 21.1 ± 3.5 kg/m^2^. Overall, the combined average weight of all participants increased from 63.6 ± 12.6 kg to 65.4 ± 13.2 kg, and BMI rose from 21.5 ± 3.5 kg/m^2^ to 22.1 ± 3.7 kg/m^2^. These findings suggest a general trend of weight and BMI gain among participants after studying abroad, potentially attributable to changes in lifestyle, dietary habits, and PA levels.

Table 2 displays the distribution of psychological factors, PA, and lifestyle characteristics among participants (*n* = 445) across different weight change groups. Regardless of gender, most factors were associated with a higher likelihood of weight gain than weight loss. While a majority of participants reported no feelings of depression, the differences between groups were not statistically significant (χ^2^ = 10.25). However, significant differences were observed in stress levels (χ^2^ = 19.97 **) and fatigue (χ^2^ = 18.12 **), with a higher proportion of participants reporting occasional stress and fatigue. For vigorous PA, significant differences were noted across activity levels (χ^2^ = 61.38 ***), with most participants falling into the optimal or no-exercise categories, and fewer engaging in high-intensity activity. A similar pattern was observed for MPA (χ^2^ = 28.82 ***), with significant differences among moderate, excessive, and no activity levels. Although most participants maintained healthy lifestyle habits, differences in SB (χ^2^ = 12.61 *) and sleep duration (χ^2^ = 18.48 *) were less pronounced compared to exercise habits and psychological factors.

Table 3 presents the results of the multinomial logistic regression analysis conducted among males, with the weight loss group serving as the reference group. Weight gain was markedly less likely with frequent stress (OR = 0.24 *, 95% CI: 0.07–0.80) and exhibited a non-significant reduction under occasional stress (OR = 0.57, 95% CI: 0.22–1.46). Weight gain was inversely related to high VPA (OR = 0.34, 95% CI: 0.09–1.31) and optimal VPA (OR = 0.68, 95% CI: 0.32–1.45) whereas optimal moderate activity significantly increased weight gain odds (OR = 1.98 *, 95% CI: 1.03–3.78), with high MPA showing a non-significant trend (OR = 1.64, 95% CI: 0.42–6.40). Weight gain was unaffected by excessive sedentary behavior (OR = 1.04, 95% CI: 0.53–2.01) but was less likely when sedentary time was low (OR = 0.59 *, 95% CI: 0.20–1.75). Weight gain continued to be less likely with frequent stress (OR = 0.26 *, 95% CI: 0.08–0.88) and showed a non-significant inverse trend for occasional stress (OR = 0.62, 95% CI: 0.24–1.62). While optimal MPA persisted as a significant risk factor (OR = 2.28 *, 95% CI: 1.16–4.49) and high MPA showed no meaningful effect (OR = 1.49, 95% CI: 0.37–6.05). Finally, short sleep duration remained a strong predictor of weight gain (OR = 2.51 *, 95% CI: 1.17–5.41), with excessive sleep still showing no effect (OR = 0.74, 95% CI: 0.25–2.15).

**Figure 1:**
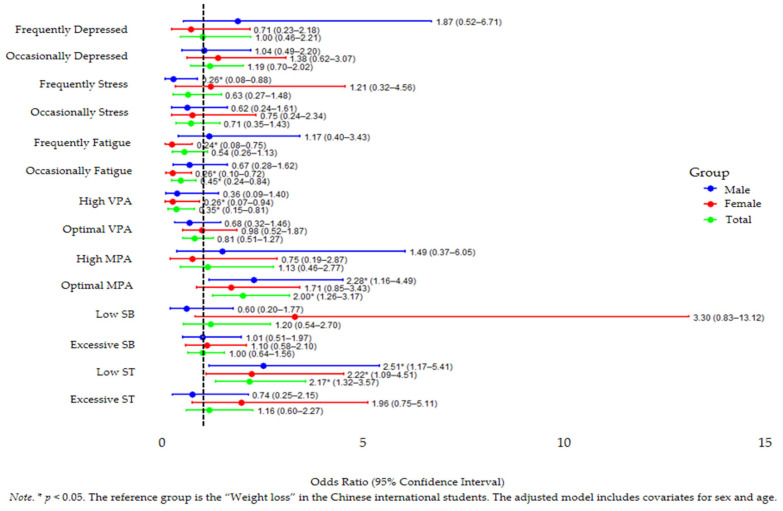
Forest Plot of the Associations Between Mental Health, Physical Activity, Sedentary Behavior, and Screen Time and Changes in Body Weight Among Chinese International Students, Adjusted age and sex for Covariates.

Table 4 and Figure 1 summarize the results of the multinomial logistic regression analysis for female participants, with the weight loss group specified as the reference group. Weight gain was markedly reduced in the presence of frequent fatigue (OR = 0.25 *, 95% CI: 0.08–0.76) and also with occasional fatigue (OR = 0.27 *, 95% CI: 0.10–0.74). Weight gain was inversely related to high vigorous physical activity (OR = 0.25 *, 95% CI: 0.07–0.91), while optimal VPA showed no significant effect (OR = 0.98, 95% CI: 0.52–1.87). Finally, weight gain odds were elevated with excessive sleep (OR = 1.94, 95% CI: 0.75–5.05) and were significantly higher with short sleep duration (OR = 2.27 *, 95% CI: 1.12–4.60). The protective effect of fatigue persisted for both frequent (OR = 0.24 *, 95% CI: 0.08–0.75) and occasional fatigue (OR = 0.26 *, 95% CI: 0.10–0.72). Lastly, excessive sleep showed no meaningful effect on weight gain (OR = 1.96, 95% CI: 0.75–5.11), and short sleep duration persisted as a significant predictor (OR = 2.22 *, 95% CI: 1.09–4.51).

## 4. Discussion

The accelerated pace of globalization has reshaped the behavioral and psychosocial environments of international students, prompting significant lifestyle changes with direct implications for health outcomes. Among these, weight change has emerged as a salient concern, driven by complex interactions between psychological health, PA, SB, and sleep duration [42,43]. Consistent with prior research [6,43], our findings demonstrate a general trend of weight gain following immigration among Chinese international students. However, the determinants of these weight changes appear to be gender-specific, highlighting the need for differentiated approaches to health management.

Psychological factors demonstrated distinct associations by gender. In males, frequent stress was inversely associated with weight gain—a finding that diverges from conventional models linking stress to overeating and adiposity. This observation is consistent with evidence that acute stress can suppress appetite (hypophagia) via CRF and noradrenergic pathways and with laboratory studies showing that men consume less under stress whereas women tend to increase intake of palatable foods [44,45]. This may reflect stress-induced hypophagia or heightened sympathetic activation [46,47]. In contrast, among females, fatigue emerged as a significant protective factor, with both frequent and occasional fatigue inversely associated with weight gain. This may suggest behavioral adaptations such as reduced energy intake or altered metabolic responses to chronic fatigue. Interestingly, depressive symptoms were not significantly associated with weight change in either sex, contrary to previous studies linking depression with altered eating behaviors and BMI fluctuations [48]. This discrepancy may stem from sample differences or cultural attitudes toward emotional expression and help-seeking behavior among East Asian populations.

Marked gender differences were also observed in the relationship between PA intensity and weight outcomes. Among males, optimal levels of MPA were significantly associated with greater odds of weight gain, consistent with the compensation hypothesis, whereby individuals increase caloric intake following exercise, thus offsetting energy expenditure. [49]. In contrast, VPA was associated with a protective, albeit non-significant, trend. Among females, however, only high levels of VPA were significantly associated with reduced odds of weight gain, suggesting that higher-intensity activity may be required to induce meaningful metabolic shifts in this group. This finding aligns with previous studies emphasizing sex-based physiological responses to exercise and underscores the importance of tailoring activity prescriptions by gender [50,51,52,53].

Contrary to several cross-sectional studies that have reported positive associations between SB and BMI [54,55], our results show no significant relationship between excessive SB and weight gain in either sex. Interestingly, low levels of SB were associated with reduced weight gain risk in males but paradoxically showed a trend toward increased risk among females, albeit non-significant. These mixed findings may reflect residual confounding, differential patterns of activity breaks, or measurement limitations in capturing intermittent movement [56]. The lack of a robust independent association reinforces the need for multidimensional analyses that control for energy intake, PA, and circadian rhythm disruption.

Sleep duration emerged as one of the most salient predictors of weight change. Short sleep was significantly associated with increased odds of weight gain in both sexes (OR = 2.67 in males; OR = 2.27 in females), corroborating previous research linking sleep deprivation to dysregulated leptin and ghrelin secretion, insulin resistance, and heightened appetite [57]. In females, excessive sleep duration also trended toward an increased risk of weight gain, suggesting a U-shaped relationship between sleep and metabolic health. This pattern may reflect an interplay between circadian disruption, mental health, and reduced energy expenditure. These findings further validate the inclusion of sleep hygiene in obesity prevention frameworks [58].

From a public health perspective, these results underscore the necessity of targeted, gender-responsive interventions. While males may benefit from strategies that emphasize sleep optimization and caloric awareness following moderate PA, female-focused interventions may be more effective if they prioritize structured high-intensity PA and fatigue-sensitive behavioral modifications. Though depression and stress did not emerge as primary predictors of weight gain in this study, their indirect effects on health behaviors and metabolic regulation should not be overlooked.

A key strength of this study lies in its gender-stratified analytic design, which elucidates nuanced pathways of risk often masked in aggregate analyses. Nonetheless, several limitations warrant consideration. Given the cross-sectional design, findings indicate associations rather than causal effects. Moreover, because weight change was defined as the difference between a pre-departure (event-anchored) baseline and a single current measure after ≥6 months in Korea, our estimates represent net change and do not identify when during university (e.g., first vs. later years) the change began. First, reliance on self-reported data introduces the potential for recall bias, social desirability bias, and misclassification. Second, reliance on self-reported data introduces the potential for recall bias, social desirability bias, and misclassification. Third, because body composition was not measured (e.g., fat mass vs. Fat free mass), we cannot rule out that observed weight gain among more active participants reflects lean mass accretion rather than increased adiposity; therefore, associations involving PA should be interpreted with caution. Finally, despite rigorous exclusion criteria and data cleaning, unmeasured confounding (e.g., diet composition, hormonal status, cultural adaptation) may still influence the observed relationships.

We acknowledge that this sign-based definition may capture very small, non-clinical fluctuations; future longitudinal work should adopt clinically interpretable thresholds (e.g., ±1.0 kg or ≥5%) and include objective monitoring and dietary assessment. For example, we cannot distinguish a third-year student who lost weight early and later regained it from one who steadily gained weight throughout the study period. Repeated measures would be required to resolve the trajectory. Finally, despite rigorous exclusion criteria and data cleaning, unmeasured confounding (e.g., diet composition, hormonal status, cultural adaptation) may still influence the observed relationships. Future longitudinal studies utilizing wearable devices and biomarker data could provide more robust causal inferences.

## 5. Conclusions

This study highlights gender-specific associations among psychological health, PA, SB, and sleep duration in determining weight change among Chinese international students. While stress and moderate PA were associated with weight change in males, fatigue and vigorous PA were more salient correlates among females. Sleep deprivation was consistently associated with weight gain across both sexes. Given the cross-sectional design, these findings indicate associations rather than causal effects. These findings reflect the broader impact of environmental and behavioral adaptation in migrant student populations and emphasize the need for integrated, tailored health promotion strategies. Practically, universities should consider sleep-hygiene programs for international students (regular sleep–wake schedules, limits on late-night screen use, and quiet hours policies). Complementary actions include sedentary break prompts on campus; accessible, supervised PA options (emphasizing moderate-intensity offerings for males and fatigue-aware pacing for females); and stress/acculturation supports (brief CBT or mindfulness workshops, peer mentoring, and language/cultural services). Policymakers, educational institutions, and public health agencies must develop holistic, gender-sensitive intervention programs that concurrently address mental health, exercise habits, and SB to mitigate the risk of weight-related comorbidities in this vulnerable population. A multidimensional framework that transcends single-factor interventions is essential for promoting metabolic health and quality of life in international student communities.

## Figures and Tables

**Table 1:** Characteristics and anthropometrics for participants in two groups (*n* = 445).

Variables			Before Studying Abroad		After Studying Abroad	
			No. (%)	Mean ± SD	No. (%)	Mean ± SD
Gender	Male		224 (50.3)		224 (50.3)	
	Female		221 (49.7)		221 (49.7)	
Anthropometrics	Male	Age (yr)	224 (50.3)	23.4 ± 2.8	224 (50.3)	23.4 ± 2.6
		Height (cm)		177.4 ± 5.7		177.4 ± 5.7
		Weight (kg)		70.0 ± 11.7		72.4 ± 12.2
		BMI (kg/m^2^)		22.2 ± 3.6		23.0 ± 3.5
	Female	Age (yr)	221 (49.7)	23.4 ± 2.6	221 (49.7)	23.4 ± 2.6
		Height (cm)		165.5 ± 5.4		165.5 ± 5.4
		Weight (kg)		56.9 ± 9.9		58.0 ± 10.7
		BMI (kg/m^2^)		20.7 ± 3.2		21.1 ± 3.5
	Total	Age (yr)	445 (100)	23.4 ± 2.7	445 (100)	23.4 ± 2.7
		Height (cm)		171.6 ± 8.2		171.6 ± 8.2
		Weight (kg)		63.6 ± 12.7		65.4 ± 13.6
		BMI (kg/m^2^)		21.5 ± 3.5		22.1 ± 3.7

*Note.* Depending on the group, the values of gender and age were expressed as frequency (*n*) and percentage (%), and the values of anthropometrics were presented as the Mean ± SD. SD: standard deviation. Body mass index (BMI) was calculated by dividing the weight (kg) by the square of the height (m^2^).

**Table 2:** Chi-Square Analysis of Psychological and Physical Activity Differences Across Weight Change Groups *(n* = 445).

Variables		Males		Females		X^2^
		Gain (%)	Loss (%)	Gain (%)	Loss (%)	
Depressed	Frequently	13 (5.8)	7 (3.1)	19 (8.6)	18 (8.1)	10.25
	Occasionally	74 (33.0)	52 (23.2)	71 (32.1)	47 (21.3)	
	Not	54 (24.1)	24 (10.7)	41 (18.6)	25 (11.3)	
Stress	Frequently	23 (10.3)	21 (9.4)	38 (17.2)	24 (10.9)	19.97 **
	Occasionally	79 (35.3)	51 (22.8)	66 (29.9)	57 (25.8)	
	Not	39 (17.4)	11 (4.9)	27 (12.2)	9 (4.1)	
Fatigue	Frequently	31 (13.8)	16 (7.1)	36 (16.3)	31 (14.0)	18.12 **
	Occasionally	74 (33.0)	53 (23.7)	63 (28.5)	52 (23.5)	
	Not	36 (16.1)	14 (6.3)	32 (14.5)	7 (3.2)	
VPA	High	10 (4.5)	12 (5.4)	5 (2.3)	11 (5.0)	61.38 ***
	Optimal	99 (44.2)	54 (24.1)	51 (23.1)	32 (14.5)	
	Insufficient	32 (14.3)	17 (7.6)	75 (33.9)	47(21.3)	
MPA	High	9 (4.0)	8 (3.6)	6 (2.7)	8 (3.6)	28.82 ***
	Optimal	86 (38.4)	37 (16.5)	55 (24.9)	25 (11.3)	
	Insufficient	46 (20.5)	38 (17.0)	70 (31.7)	57 (25.8)	
SB	Excessive	48 (21.4)	35 (15.6)	62 (28.1)	48 (21.7)	12.61 *
	Low	9 (4.0)	8 (3.6)	12 (5.4)	4 (1.8)	
	Normal	84 (37.5)	40 (17.9)	57 (25.8)	38 (17.2)	
Sleep Time	Excessive	8 (3.6)	11 (4.9)	17 (7.7)	10 (4.5)	18.48 *
	Low	46 (20.5)	12 (5.4)	46 (20.8)	20 (9.0)	
	Normal	87 (38.8)	60 (26.8)	68 (30.8)	60 (27.1)	

*Note.* Cell entries are *n* (%) by sex. Percentages use the sex-specific total sample as the denominator. The Pearson χ^2^ shown tests the independence between weight-change group (gain vs. loss) and the listed categorical factor in the combined sample. Exact *p*-values are reported; significance markers: *** *p* < 0.001, ** *p* < 0.01, * *p* < 0.05. “Males/Females” denote sex-stratified counts; the χ^2^ assesses the overall association, not a between-sex comparison.

**Table 3:** Comparative analysis of health-related factors across weight change groups, results of multinomial logistic regression analysis for males (n = 224).

Variables		Unadjusted			Adjusted		
		OR	95% CI		OR	95% CI	
			Lower	Upper		Lower	Upper
Depressed	Frequently	1.84	0.52	6.45	1.87	0.52	6.71
	Occasionally	1.02	0.48	2.15	1.04	0.49	2.20
	None						
Stress	Frequently	0.24 *	0.07	0.8	0.26 *	0.08	0.88
	Occasionally	0.57	0.22	1.46	0.62	0.24	1.62
	None						
Fatigue	Frequently	1.24	0.43	3.61	1.17	0.40	3.43
	Occasionally	0.69	0.29	1.66	0.67	0.28	1.62
	None						
VPA	High	0.34	0.09	1.31	0.36	0.09	1.4
	Optimal	0.68	0.32	1.45	0.68	0.32	1.46
	Insufficient						
MPA	High	1.64	0.42	6.40	1.49	0.37	6.05
	Optimal	1.98 *	1.03	3.78	2.28 *	1.16	4.49
	Insufficient						
SB	Excessive	1.04	0.53	2.01	1.01	0.51	1.97
	Low	0.59 *	0.2	1.75	0.60	0.21	1.78
	Normal						
ST	Excessive	0.72	0.25	2.06	0.74	0.25	2.15
	Low	2.67	1.25	5.7	2.51 *	1.17	5.41
	Normal						

*Note*. * *p* < 0.05. VPA: vigorous physical activity; MPA: moderate physical activity; SB: sedentary behaviour; ST: sleep time. OR: Odds Ratio. The reference group is the males in the weight loss group. The adjusted model includes covariates for sex and age.

**Table 4:** Comparative analysis of health-related factors across weight change groups, results of multinomial logistic regression analysis for females (*n* = 221).

Variables		Unadjusted			Adjusted		
		OR	95% CI		OR	95% CI	
			Lower	Upper		Lower	Upper
Depressed	Frequently	0.69	0.22	2.11	0.71	0.23	2.18
	Occasionally	1.38	0.62	3.07	1.38	0.62	3.07
	None						
Stress	Frequently	1.19	0.32	4.46	1.21	0.32	4.56
	Occasionally	0.72	0.23	2.25	0.75	0.24	2.34
	None						
Fatigue	Frequently	0.25 *	0.08	0.76	0.24 *	0.08	0.75
	Occasionally	0.27 *	0.10	0.74	0.26 *	0.10	0.72
	None						
VPA	High	0.25 *	0.07	0.91	0.26	0.07	0.94
	Optimal	0.98	0.52	1.87	0.98	0.52	1.87
	Insufficient						
MPA	High	0.73	0.19	2.81	0.75	0.19	2.87
	Optimal	1.68	0.84	3.37	1.71	0.85	3.43
	Insufficient						
SB	Excessive	1.14	0.6	2.15	1.11	0.58	2.10
	Low	3.34	0.84	13.25	3.30	0.83	13.12
	Normal						
ST	Excessive	1.94	0.75	5.05	1.96	0.75	5.11
	Low	2.27 *	1.12	4.6	2.22 *	1.09	4.51
	Normal						

*Note*. * *p* < 0.05. VPA: vigorous physical activity; MPA: moderate physical activity; SB: sedentary behaviour; ST: sleep time. OR: Odds Ratio. The reference group is the females in the weight loss group. The adjusted model includes covariates for sex and age.

## Data Availability

The datasets used and analyzed during the current study are available from the corresponding author upon reasonable request.

## Abbreviations

The following abbreviations are used in this manuscript:

PA	Physical activity
VPA	Vigorous Physical Activity
MPA	Moderate Physical Activity
SB	Sedentary Behavior
ST	Sleep Time
OR	Odds Ratio
CI	Confidence Interval
BMI	Body mass index
